# Editorial: Advances and controversies in ischemic stroke management: from prevention to diagnosis and acute treatment

**DOI:** 10.3389/fneur.2026.1687915

**Published:** 2026-02-13

**Authors:** Lucio D'Anna, Giovanni Merlino, Raffaele Ornello, Matteo Foschi

**Affiliations:** 1 Department of Stroke and Neuroscience, Charing Cross Hospital, Imperial College London NHS Healthcare Trust, London, United Kingdom; 2 Department of Brain Sciences, Imperial College London, London, United Kingdom; 3 Clinical Neurology, Udine University Hospital and Department of Medicine (DMED), University of Udine, Udine, Italy; 4 Struttura Operativa Semplice Dipartimentale (SOSD) Stroke Unit, Department Head, Neck, and Neurosciences, Udine University Hospital, Udine, Italy; 5 Department of Biotechnological and Applied Clinical Sciences, University of L'Aquila, L'Aquila, Italy; 6 Department of Neurosciences, Stroke Unit - Neurology Unit, S. Maria delle Croci Hospital, AUSL Romagna, Ravenna, Italy

**Keywords:** stroke, acute ischemia-reperfusion, thrombectomy, recanalization, biomarkers

Ischemic stroke remains a leading cause of death and long-term disability worldwide. The clinical and scientific landscape is rapidly evolving, with ongoing advances in prevention, diagnosis, acute treatment, and prognostication. The articles collected in this Research Topic address multiple aspects of stroke care, providing novel evidence across the continuum of management.

In terms of diagnosis and early recognition, several studies focused on improving the accuracy and timeliness of ischemic stroke detection. Jin et al. combined clinical examination with diffusion-weighted imaging to differentiate posterior circulation infarctions from mimics, underscoring the role of integrated clinical–radiological assessment. Schaefer et al. explored plasma biomarkers for pre-hospital stroke identification, supporting the feasibility of biochemical triage tools. Quantitative imaging approaches were also investigated, including the evaluation of the RAPID non-contrast CT stroke platform for large vessel occlusion and intracranial hemorrhage detection, and dual-phase CT angiography for rapid prognostic stratification in patients with large ischemic regions. Additional contributions examined the predictive value of the modified Prehospital Acute Stroke Severity scale in posterior circulation occlusion and the role of advanced MRI signatures in distinguishing posterior circulation infarction from vestibular neuritis in acute vestibular syndrome.

Regarding acute treatment, multiple articles addressed refinements in reperfusion strategies. Lin, Zhao et al. demonstrated the prognostic relevance of the Prominent Cortical Vein sign on post-thrombectomy outcomes. Pei et al. identified clinical and imaging factors linked to poor prognosis after thrombectomy, while Sun et al. developed a nomogram to predict futile recanalization. Comparative effectiveness research included analyses of combined stent retriever plus aspiration vs. stent retriever alone, and meta-analyses comparing general anesthesia to conscious sedation during thrombectomy. The efficacy of endovascular treatment beyond 24 h, guided by CT perfusion, was examined, as well as treatment of anterior cerebral artery occlusion and basilar artery occlusion. Pharmacological adjuncts were also explored, including PCSK9 inhibitors in mechanical thrombectomy patients, tirofiban in non-thrombectomy cases, and low-dose or no heparinization during cerebral angiography. Workflow optimization studies assessed time-saving models for acute treatment in China and strategies for reducing onset-to-door and onset-to-groin times.

With respect to secondary prevention, research emphasized risk factor stratification and targeted therapy. Lin, Si et al. reported that the ApoB/ApoA-I ratio predicts recurrent stroke, while Hu et al. used Mendelian randomization to investigate links between chronic rhinosinusitis and stroke risk. Other genetic epidemiology studies examined causal associations between inflammatory bowel disease and ischemic stroke, bone mineral density and stroke outcomes, and fatigue as a mediator of post-stroke prognosis. Several contributions focused on lipid-related indices, such as the triglyceride–glucose index and triglyceride-to-HDL ratio, as predictors of early neurological deterioration. Antiplatelet therapy optimization was addressed in propensity-matched analyses of cilostazol-based dual therapy and systematic reviews comparing clopidogrel plus aspirin for varying treatment durations in symptomatic intracranial stenosis. Trials and cohort studies also evaluated secondary prevention strategies in atrial fibrillation, including the ACE2L score for predicting AF in cryptogenic stroke and the ATIS-NVAF protocol for optimal antithrombotic therapy.

In the area of biomarkers and prognosis, a substantial number of studies investigated circulating and imaging markers to refine prognostic assessment. Song et al. demonstrated that elevated inflammatory indices, particularly the neutrophil-to-lymphocyte ratio, are independently associated with unfavorable functional outcomes, while Jiang et al. evaluated a comprehensive panel of circulating inflammatory biomarkers to develop prognostic models for short-term outcomes in acute ischemic stroke. Sun et al. reported an association between higher baseline bilirubin and favorable outcomes, and other studies linked the eosinophil-to-neutrophil ratio, fibrinogen levels, and the hemoglobin-to-red cell distribution width ratio to functional recovery. Imaging biomarkers included lesion core extent, ASPECTS progression, and vascular hyperintensity–DWI mismatch for late-window thrombectomy selection. Prognostic models were developed for specific complications such as ventilator-associated pneumonia and malignant cerebral edema, and for predicting symptomatic intracranial hemorrhage based on admission glucose or the total cholesterol-to-HDL ratio after successful thrombectomy.

Finally, at the level of public health and systems of care, several contributions extended the focus beyond individual patient management to healthcare systems and population-level interventions. Sese et al. highlighted the importance of public awareness campaigns for stroke recognition, particularly in underserved communities. Lee et al. provided a systematic review of registered prevention programmes, mapping global strategies. Other studies addressed disparities in access to endovascular therapy, including geographic, socioeconomic, and temporal (“weekend effect”) factors. Workflow and organizational improvements were explored in telestroke networks, including evaluations from Colombia and rural healthcare systems. Further work examined the integration of artificial intelligence into stroke pathways to improve diagnostic accuracy and streamline acute care delivery.

In summary, the studies collected in this Research Topic provide a broad and multifaceted overview of current advances and ongoing debates in ischemic stroke management. The evidence spans early diagnosis, acute treatment optimization, targeted secondary prevention, biomarker discovery, and system-level strategies, reflecting the complexity of improving outcomes in this heterogeneous condition. The integration of translational research, clinical trials, and real-world data offers a rich foundation for future investigation and implementation in diverse healthcare settings.

